# The Prediction of Human Abdominal Adiposity Based on the Combination of a Particle Swarm Algorithm and Support Vector Machine

**DOI:** 10.3390/ijerph17031117

**Published:** 2020-02-10

**Authors:** Xiue Gao, Wenxue Xie, Shifeng Chen, Junjie Yang, Bo Chen

**Affiliations:** 1 College of Information Engineering, Lingnan Normal University, 29th Cunjin Road, Chikan Zone, Zhanjiang 524048, Guangdong, China; gaoxiue510@126.com (X.G.); xiewenxue12345@163.com (W.X.) chens@dlmu.edu.cn (S.C.); jjyang2013@163.com (J.Y.); 2 College of Information Engineering, Dalian University, 10th Xuefu street, Dalian Economic and Technological Development Zone, Dalian 116622, Liaoning, China

**Keywords:** human abdominal adiposity, selection of characteristic parameters, particle swarm algorithm, improved support vector machine

## Abstract

*Background*: Abdominal adiposity is an important risk factor of chronic cardiovascular diseases, thus the prediction of abdominal adiposity and obesity can reduce the risks of contracting such diseases. However, the current prediction models display low accuracy and high sample size dependence. The purpose of this study is to put forward a new prediction method based on an improved support vector machine (SVM) to solve these problems. *Methods*: A total of 200 individuals participated in this study and were further divided into a modeling group and a test group. Their physiological parameters (height, weight, age, the four parameters of abdominal impedance and body fat mass) were measured using the body composition tester (the universal INBODY measurement device) based on BIA. Intelligent algorithms were used in the modeling group to build predictive models and the test group was used in model performance evaluation. Firstly, the optimal boundary C and parameter gamma were optimized by the particle swarm algorithm. We then developed an algorithm to classify human abdominal adiposity according to the parameter setup of the SVM algorithm and constructed the prediction model using this algorithm. Finally, we designed experiments to compare the performances of the proposed method and the other methods. *Results*: There are different abdominal obesity prediction models in the 1 KHz and 250 KHz frequency bands. The experimental data demonstrates that for the frequency band of 250 KHz, the proposed method can reduce the false classification rate by 10.7%, 15%, and 33% in relation to the sole SVM algorithm, the regression model, and the waistline measurement model, respectively. For the frequency band of 1 KHz, the proposed model is still more accurate. (4) *Conclusions*: The proposed method effectively improves the prediction accuracy and reduces the sample size dependence of the algorithm, which can provide a reference for abdominal obesity.

## 1. Introduction

As the number of fat people increases, obesity has become a worldwide epidemic. The number of patients with obesity has exceeded the number of patients who have contracted infectious diseases or who suffer from undernutrition. It constitutes a severe menace to human health [1], as overweight or obesity can easily cause high blood pressure [2]. Unfortunately, the populations of patients with obesity are increasing every year in many countries. It is well known that the main manifestation of obesity is abdominal obesity which is an important factor leading to chronic diseases [3]. Especially, there are populations with hidden abdominal obesity. In other words, their body size does not show obesity but they have high levels of visceral fat. It is difficult to directly detect the patients with hidden abdominal obesity by using methods such as BMI and waist circumference. What is more, these populations are more inclined to contract cardiovascular diseases (CVD) or diabetes [4,5]. Therefore, the abdominal adiposity prediction is beneficial to disease prevention and the mathematical model method can predict abdominal obesity more effectively. The researchers have studied methods for the prediction of abdominal fat area/content and abdominal adiposity. The primary methods are image inspection and bioelectrical impedance analysis (BIA).

The image inspection techniques for predicting abdominal adiposity and obesity include: computed tomography (CT), magnetic resonance imaging (MRI), ultrasound, and dual-energy X-ray absorptiometry (DXA). Han [6] analyzed the statistics of the abdominal fat distribution, the hepatic lipid content, and obesity and found that quantitative CT can be used to evaluate the abdominal fat distribution of an obese subject which can be used to predict the risk of obesity. Hongxin [7] measured anthropometric indices such as abdominal height, waistline, body mass index (BMI), and height by using CT found that the abdominal height can be used to effectively predict abdominal adiposity. Hung [8] determined the visceral fat of the elderly by measuring the living space during the abdominal ultrasound examination. The method is practical but the prediction accuracy is not high. In addition, researchers are more inclined to build mathematical models to make predictions, which is more effective and convenient. Pintér [9] developed a regression equation for the prediction of the total abdominal fat area, subcutaneous fat area, and visceral fat area based on the anthropometric characteristics and CT image. The results demonstrated the equation is more effective in prediction but there exist certain systematic errors. Chen [10] developed a CT-based regression equation to estimate the visceral fat area. The equation can rapidly and accurately estimate the visceral fat area. Liu [11] developed MRI-based regression equations to predict the abdominal and pelvic fat respectively for men and women. However, the operation is difficult and the expensive. Rina [12] developed a multivariate regression equation to predict the volume of the abdominal visceral adipose tissues, based on MRI measurements such as the waistline. Sun [13] developed a MRI based regression equation to predict abdominal adiposity and a novel shape descriptor of 3-D human body image. Their experiments demonstrated that the method can effectively predict the abdominal adiposity quantified by MRI. Rolfe [14] improved the accuracy of a regression equation predicting the visceral fat and the abdominal subcutaneous fat of the elderly subjecting to ultrasound measurement. Manios [15] established a mathematical model to estimate postmenopausal Caucasian women’s AFM% based on the data obtained by DXA. Their experiments demonstrated that the model is simple, practical, and inexpensive. But the method causes radiation damages to the human body.

BIA is widely used in researches due to the merits such as non-invasiveness, convenience, and low costs. Eickemberg [16] predicted the visceral fat of adults and old people by BIA and found that BIA has satisfactory sensitivity and specificity. Lu [17] predicted the abdominal visceral fat area of Chinese by leg-to-leg BIA (LBIA) and found that LBIA has limited prediction precision. Gómez-Ambrosi [18] used the abdominal BIA to measure the visceral adipose tissues and found that it is a good substitution for the imaging-based prediction of the visceral adiposity. What is more, the mathematical model based on BIA technology has better prediction effect. Wang [19] put forward a model to predict the abdominal fat area based on BIA. The simulations demonstrated that the model can effectively predict the abdominal fat area. Zhu [20] developed a model combining BIA and the wavelet neural network cloud model to predict human body visceral fat area. The model is more effective in prediction than a general regression based model.

In summary, mathematical models based on these two types of technology have a better prediction effect. However, imaging has obvious shortcomings, such as radiation, high cost, and limited testing space. BIA, due to the merits of non-invasiveness, non-radiation, convenience, and low costs, has gradually been applied to researches on human abdominal adiposity. However, most of the current BIA based methods depend on specific samples and are difficult to solve the problem of small sample size, whose prediction accuracy is unsatisfactory.

In this paper, we put forward a method of abdominal adiposity prediction based on BIA and improved support vector machine (iSVM), in order to improve prediction accuracy and solve the problem that the model is affected by the sample size. The main contributions include:

(1) The application of particle swarm algorithm (PSA) to optimize the parameters of SVM, which obtains the improved SVM (iSVM);
(2) An abdominal adiposity classification model is developed using iSVM to process the data obtained from BIA;
(3) The simulation experiments were designed to evaluate the prediction accuracy and effectiveness of abdominal adiposity classification in two frequency bands.

## 2. Materials and Methods

### 2.1. Subjects

The study was approved by the Ethics Review Board of Lingnan Normal University. Ninety eight (98) men and 102 women were recruited for the study via posted notices and word-of-mouth and informed consent was obtained. The sample consisted of 200 healthy Chinese who were instructed to fast for 4 h and avoid heavy exercise, alcohol, or caffeine for 10 h prior to the visit. Participants have three clinical characteristics: normal, overweight and obese. They were measured human physiological parameters, including BMI, weight, body fat mass, and human electrical impedance, on a human body composition tester based on BIA technology.

### 2.2. Selection of the Key Characteristics of Human Abdominal Adiposity for the Prediction Model

The first step of constructing the prediction model is to select the key characteristics of human abdominal adiposity. We used the body subdivision eight-segment impedance model [21] to obtain the four parameters of abdominal impedance $R_{1}\sim R_{4}$. Let T=(O,F,C) to be the dataset of body composition. We classified the important body composition parameters, such as weight, height, age, sex, abdominal impedance, into the first kind of characteristics. Let the inverse ($1/R_{i}$), the square ($R_{i}{}^{2}$), the product ($R_{i}R_{j}$) of the abdominal segmental impedances to be the second kind of characteristics. We included $R_{1},$ $R_{2},$ $R_{3},$ $R_{4},$ A, H, W from the first kind of characteristics and $1/R_{1},$ $1/R_{2},$ $1/R_{3},$ $1/R_{4},$ $R_{1}R_{2},$ $R_{1}R_{3},$ $R_{1}R_{4},$ $R_{2}R_{3},$ $R_{2}R_{4},$ $R_{3}R_{4},$ $R_{1}{}^{2},$ $R_{2}{}^{2},$ $R_{3}{}^{2},$ $R_{4}{}^{2},$ from the second kind of characteristics in a set called the original characteristic parameters, denoted by $F=\{f_{1},f_{2},\cdot \cdot \cdot ,f_{m}\}$. We chose Body Fat Mass (BFM) to be the body composition index.

**Figure 1:**
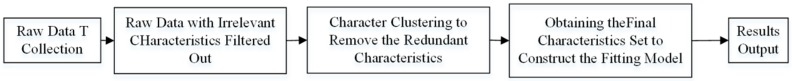
Selection of the characteristic parameters of the abdominal obesity prediction model.

We selected the key characteristics by using a human characteristics selection algorithm combining filtering and clustering [22]. We first used the Filter characteristics selection algorithm to remove those characteristics irrelevant to the body composition (BFM). We then used the M-Chameleon clustering method to remove the redundant characteristics. Finally, the characteristics most suitable to predicting the abdominal adiposity were obtained. The whole process is illustrated by Figure 1.

As mentioned above, the data were included in the set T=(O,F,C), where the sampling data were in the data samples set $O=\{o_{1},o_{2},\cdot \cdot \cdot ,o_{n}\}$, the set of the selected characteristics were in the set $F=\{f_{1},f_{2},\cdot \cdot \cdot ,f_{m}\}$, and the human body composition classes were in the set $C=\{c_{1},c_{2},\cdot \cdot \cdot ,c_{n}\}$. Because BFM is often used to evaluate adiposity in the abdomen, the BFM body composition index C was used as an input. By characteristic filtering, we deleted those characteristics that are irreverent to the body composition index C. We thus obtained the initial set of characteristics $F^{\prime }=\{f_{1},f_{2},\cdot \cdot \cdot ,f_{h}\}$ and the initial dataset $T^{\prime }=(O,F^{\prime },C)$. We then used the clustering algorithm to pre-process the data. Each cluster is updated by removing those distant characteristics. In the final clusters, we selected those characteristics whose relative interconnection and relative proximity with the center are either smaller or equal to 90%. The selected characteristics were included in the best candidate characteristics set $X=\{f_{1},f_{2},\cdot \cdot \cdot ,f_{l}\}$.

### 2.3. A iSVM Based Algorithm Classifying Abdominal Adiposity

SVM is a classifier with sparsity and robustness which uses hinge loss function to calculate empirical risk and adds regularization terms to the solution system to optimize structural risk and its decision boundary is the maximum margin hyperplane solved for the learning sample. More importantly, SVM can perform non-linear classification by the kernel method, which is one of the common kernel learning methods. Therefore, the classification model is based on the SVM using RBF as the kernel function in this paper. The range of the boundary parameter C depends on the range of tolerable error, which is the mean of the sampling error and the structural risk. The range of the kernel parameter depends on the range of the training samples. At present, the globally optimal kernel parameter is obtained by cross-validation (CV), which requires a lot of time to achieve high prediction accuracy. We thus decided to optimize the SVM parameters by the PSA.

#### 2.3.1. Optimization of the SVM Parameters by the Particle Swarm Algorithm

Particle swarm algorithm is a random search algorithm based on swarm cooperation, which is developed by simulating the foraging behavior of bird swarms. The PSA reflects social sharing of information. The to-be-optimized parameters are called “particles,” which constitute a population $X=(X_{1},\cdots ,X_{n})$. As searching starts in a D-dimensional space, the position of the i-th particle is denoted by $X_{i}={\left(X_{i1},\cdots ,X_{iD}\right)}^{T}$. Every particle can remember its best position thus far: $gbest_{}=(g_{1},\cdots ,g_{D})$. For the i-th particle, the best position along its trajectory is denoted by $pbest_{i}=(p_{i1},\cdots ,p_{iD})$. To determine the movement of a particle in the D-dimensional space, we determine the particle’s direction by adding a position and velocity to the particle. The velocity of the i-th particle is represented by $V_{i}=(V_{i1},\cdots ,V_{iD})$, which iterates throughout the optimization according to:

(1) $$V_{id}^{k}=\omega V_{id}^{k-1}+c_{1}r_{1}(pbest_{id}-X_{id}^{{}^{k-1}})+c_{2}r_{2}(gbest_{d}-X_{id}^{{}^{k-1}})$$

where i=1,2,⋯,m; d=1,2,⋯,D; k is the number of iterations; $V_{id}^{k}$ represents the d-th dimension component of the velocity vector of the particle i at the k-th iteration. Here $c_{1},c_{2}$ represent the accelerations used to adjust the particle’s largest learning step; $r_{1},r_{2}$ are two random numbers in the range of [0,1] to enhance randomness of the search. Here ω is a non-negative number representing the inertia weight, which is used to adjust the domain of searching and to balance the tendency between local optimization and global optimization. It is iteratively updated according to:

(2) $$\omega (k)=\omega_{start}-(\omega_{start}-\omega_{end})(T_{\max}-k)/T_{\max}$$

where $\omega_{start}$ is the initial inertia weight, $\omega_{end}$ is the final inertia weight, $T_{\max}$ is the total number of iterations. As $V_{id}^{k}$ is updated, the position $X_{id}^{k}$ is updated according to:

(3) $$X_{id}^{k}=X_{id}^{{}^{k-1}}+V_{id}^{k-1}$$

#### 2.3.2. The Algorithm

**Figure 2:**
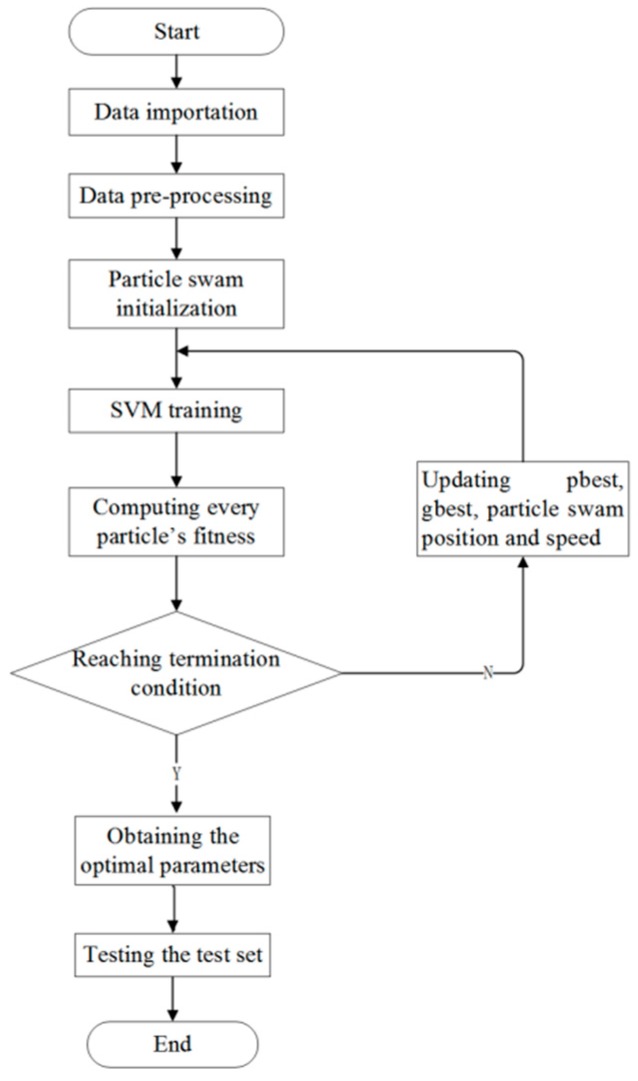
iSVM classification based on the particle swarm optimization.

In the training of iSVM, every parameter of the training samples are taken from the selected characteristic parameters in Section 2.2. The steps are as follows (see also Figure 2).

*Step 1*: Import and then normalize the data. Mix the data sampled from the obesity, overweight, and normal populations. From the mixed data randomly pick 100 data to form the training set and another 100 to form the test set.

*Step 2*: Initialize the particle swarm. Let the number of particles be 100, the same as the number of training samples ($X_{1},\cdots ,X_{100}$). The parameter values are: $\omega_{start}=0.9$, $\omega_{end}=0.4$, $c_{1},c_{2}$ = 2, $V\in [2^{-2},2^{4}]$, $T_{\max}$ = 80 [23,24].

*Step 3*: Model training in the space of the 100 samples.

*Step 4*: Check whether or not the desired accuracy has been achieved or the maximal number of iterations has been reached. If not, go to Step 5. If yes, find the optimized boundary parameter C and gamma. Choose a practical integer value for C in the range [1,4]; one practical value for gamma in the range [1,20]. Search for the optimal value of the parameter gamma in the particle swarm with the best speed. Finally the test set is used for the testing.

*Step 5*: Update the position and velocity of the particles. Update gbest and pbest. Construct a new optimal SVM model. Then go to Step 4.

## 3. Results

The experimental data consisted of the body composition data and the characteristic parameters of subjects of different sex, height, weight and age groups. For the two frequency bands 1 KHz and 250 KHz, we selected in total 200 individuals from the normal, obesity (BMI ≥28), overweight (BMI ≥24) groups. The BMI classification was based on the Chinese standard. From the 200 total, we randomly selected 100 to construct the training set and the remaining 100 constituted the test set. We constructed the classification model by letting the optimal C and gamma be 2 and 8, respectively. The simulation program was written by the R language.

### 3.1. Classification by iSVM

Table 1 lists the characteristics of the training set and the test set, which were screened in the two frequency bands 1 KHz and 250 KHz. For each of the two frequency band (1 KHz and 250 KHz), we modeled the selected characteristics by using the iSVM. We then fed the 100 test samples into the model for the classification.

**Figure 3:**
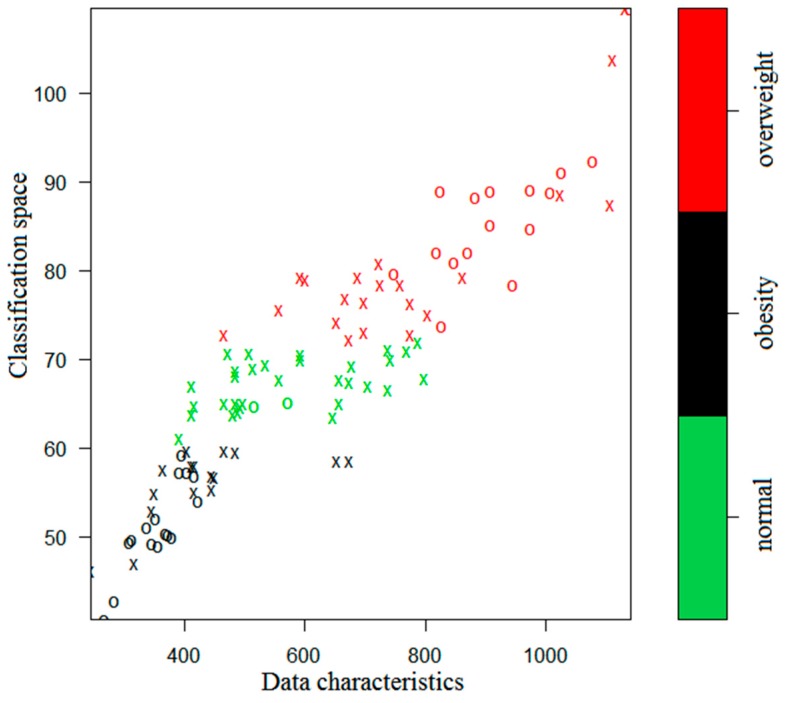
Classification for the 1 KHz case by the iSVM.

**Figure 4:**
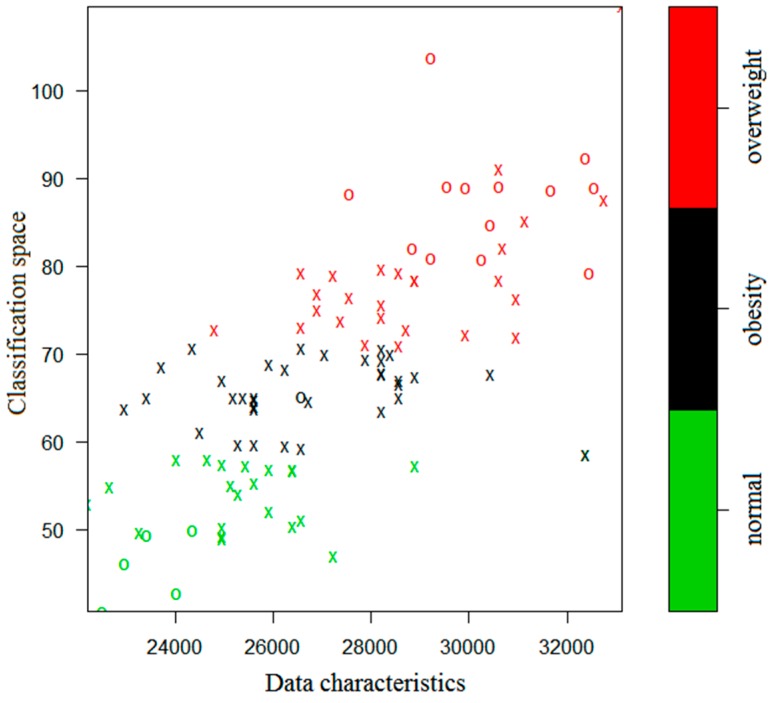
Classification for the 250 KHz case by the iSVM.

The simulation results are presented in Figure 3 and Figure 4, where the green, black, and red colors indicate the normal, obesity, and overweight subjects, respectively.

In Figure 3 and Figure 4, an X or O represents a single sample. The X’s together naturally partition the classification plane. They are thus called the support vectors. The O’s represent the other samples. From the figures one sees that the iSVM model can well distinguish the three kinds of data according to the colors. If there were only two characteristic parameters, then the classification could be done in a two-dimensional space. However, the 1 KHz and 250 KHz samples are multi-dimensional and they have 10 and 9 dimensional characteristic parameters, respectively. Therefore, the classified samples have different dimensionality.

### 3.2. Accuracy of the Prediction

To quantify the prediction accuracy, the actual classification of the obesity, overweight, and normal weight test data and the corresponding predicted classification are listed in Table 2 for a direct comparison.

To compare the accuracy of the sole SVM versus the iSVM model, we classified the prediction data several times, with the data summarized in Table 3.

One sees that for the 250 KHz frequency band, there exists considerable classification error by using the sole SVM model, which necessitates PSA to obtain more optimized boundary parameter C. In other words, the iSVM can significantly reduce the mistakenly classified samples.

### 3.3. Model Comparison

Waistline measurement and regression analysis are the two conventional methods of obesity measurement. To verify effectiveness of the present method, we applied all the three methods to the test sets, analyzed the results, and compared the three methods. The results are described as follows.

Model 1 is based on the subjects’ waistline measurement. The obesity status inferred from the measurement data is then compared with the medical measurement standard. The three methods’ time consumptions have some difference, because they depend on the level of operational skills. They are thus irrelevant to the present research and are not considered in the following.

Model 2 is based on the SPSS predicting regression model with the parameter values α=0.05,β=−89,γ=−0.04,δ=−24.66. One uses the linear regression equations obtained by SPSS to perform comparative analysis.

Model 3 uses the present iSVM to develop abdominal adiposity classification model. The training samples include the selected characteristic parameters in the frequency bands of 1 KHz and 250 KHz.

To determine which model is the most accurate, we calculated the root mean square (RMS) errors of all the three models, namely the square root of the sum of the multiple square errors. The RMS prediction errors of the three models in the frequency bands 1 KHz and 250 KHz are presented in Table 4 and Table 5, respectively. The classification results of the three models in the frequency bands 1 KHz and 250 KHz are presented in Table 6 and Table 7, respectively.

From the tables one sees that the RMS error of the prediction by the iSVM is apparently smaller than that by the traditional regression model and the classification error of the iSVM method is apparently smaller than the simple waistline measurement method. The time consumption of iSVM is a little more, due to the computation and training that were necessary to improve the accuracy.

## 4. Discussion

In order to obtain more effective prediction than traditional methods such as waist measurement and BMI, image detection and BIA technology are mainly used to predict abdominal obesity. However, there are some obvious disadvantages to image detection technology. The use of CT is particularly widespread, but the radiation does harm to the human body to a certain degree. MRI has no radiation problem, but it is expensive and has site-specific constraints. Ultrasound imaging is non-invasive and cheap, but its diagnosis precision depends on operating experience and equipment performance. X-ray imaging is simple and inexpensive, but it also has the radiation problem. Therefore, we chose to use BIA technology for abdominal prediction research.

The proposed model based on BIA technology has the advantages of harmlessness to humans, convenience and low cost. What’s more, in order to solve the problem that it is difficult to predict invisible obesity directly, we considered the abdominal electrical impedance as a predictor. Using abdominal impedance as a variable in the prediction model can more accurately predict abdominal obesity because the value of abdominal impedance changes as it becomes fatter. From the comparison with the waist circumference model, the proposed model has higher prediction accuracy, which can more effectively predict hidden obesity.

It is well known that support vector machine is often used to solve small sample size problems and PSA is an optimization algorithm. Therefore, we suggested a prediction method based on the combination of particle swarm algorithm and support vector machine for obtaining a prediction model suitable for small sample size and high accuracy. In comparison with separate support vector machine model and traditional regression model, the proposed model shows better prediction results. The reasons why the single SVM model and the traditional regression model show poor performance are that the single SVM model lacks optimized parameters and the sample size for the traditional regression model is insufficient. Accordingly, the method in this paper offers great advantages in terms of safety, cost, hidden abdominal obesity prediction, small sample size prediction and prediction accuracy.

## 5. Conclusions

In this paper, we put forward a new model of classifying abdominal adiposity. The parameters of the SVM, which uses the RBF kernel functions, were optimized by the PSA so that the SVM can achieve a higher modeling accuracy. The experimental results demonstrate that the present model has high accuracy and can effectively predict abdominal adiposity. What is more, the method in this paper has the advantages of more effective prediction effects, radiation-free, cheapness and convenience. Therefore, the present study has provided a new model for the practical abdominal adiposity prediction. Finally, a combination of image detection technology and BIA technology will be considered to predict abdominal obesity in the future research work.

## Figures and Tables

**Table 1:** The filtered and clustered characteristic parameters.

Frequency Band of Impedance	Number of Characteristics	Characteristic Set
1 KHz	10	$W,R_{4},R_{2}R_{4},R_{3}R_{4},R_{2},1/R_{3},1/R_{2},H^{2}/R_{1},H^{2}/R_{3},H^{2}/R_{2}$
250 KHz	9	$R_{2},R_{4},H,A,W,1/R_{2},R_{1}R_{4},R_{2}R_{4},H^{2}$

**Table 2:** Classification of the test data with the iSVM.

Frequency Band	Obesity (Actual/Predicted)	Overweight (Actual/Predicted)	Normal (Actual/Predicted)
1 KHz	33/33	34/34	32/32
250 KHz	28/27	37/37	35/35

**Table 3:** Comparison of the classification accuracy in the two frequency bands.

Frequency Band	Error Rate of SVM Classification	Error Rate of SPA Optimized SVM Classification
1 KHz	0	0
250 KHz	0.1,176,471	0.0100

**Table 4:** RMS prediction errors of the three models in the 1 KHz frequency band.

Model	RMS	Time Consumed/s
1	0.23	-
2	0.04	256
3	0	276

**Table 5:** RMS prediction errors of the three models in the 250 KHz frequency band.

Model	RMS	Time Consumed/s
1	0.35	-
2	0.17	344
3	0.02	356

**Table 6:** The classification results of the models in the 1 KHz frequency band.

Model	Obesity (Actual/Predicted)	Overweight (Actual/Predicted)	Normal (Actual/Predicted)
1	33/25	34/31	32/28
2	33/27	34/34	32/34
3	33/33	34/34	32/32

**Table 7:** The classification results of the models in the 250 KHz frequency band.

Model	Obesity (Actual/Predicted)	Overweight (Actual/Predicted)	Normal (Actual/Predicted)
1	28/21	37/33	35/25
2	28/28	37/35	35/34
3	28/27	37/37	35/35
